# Using several pseudo amino acid composition types and different machine learning algorithms to classify and predict archaeal phospholipases 

**DOI:** 10.22099/mbrc.2023.47756.1845

**Published:** 2023

**Authors:** Nour Samman, Hassan Mohabatkar, Parisa Rabiei

**Affiliations:** Department of Biotechnology, Faculty of Biological Science and Technology, University of Isfahan, Isfahan, Iran

**Keywords:** Archaea, Phospholipases, Machine learning, Chou’s PseAAC

## Abstract

Phospholipases, as important lipolytic enzymes, have diverse industrial applications. Regarding the stability of extremophilic archaea’s proteins in harsh conditions, analyses of unusual features of their proteins are significantly important for their utilization. This research was accomplished to *in silico* study of archaeal phospholipases’ properties and to develop a pioneering method for distinguishing these enzymes from other archaeal enzymes via machine learning algorithms and Chou’s pseudo-amino acid composition concept. The non-redundant sequences of archaeal phospholipases were collected. BioSeq-Analysis sever was used with Support Vector Machine (SVM), Random Forests (RF), Covariance Discrimination (CD), and Optimized Evidence-Theoretic K-nearest Neighbor (OET-KNN) as powerful machine learnings algorithms. Also, different Chou’s pseudo-amino acid composition modes were performed and then, 5-fold cross-validation was applied to the sequences. Based on our results, the OET-KNN predictor, with 96% accuracy, yields the best performance in SC-PseAAC mode by 5-fold cross-validation. This predictor also achieved very high values of specificity (95%), sensitivity (96%), Matthews’s correlation coefficient (0.92), and accuracy (96%). The present investigation yielded a robust anticipatory model for the archaeal phospholipase prediction utilizing the tenets PseAAC and OET-KNN machine learning algorithm.

## INTRODUCTION

Relying on comparative genomics and rRNA-based phylogenetic trees, the archaea are introduced as the third domain of life following bacteria and eukarya [1, 2]. Archaea live in harsh environments including high temperature, high osmotic pressure, and extreme pH. As a result of tolerance to these excessive surroundings where other proteins would be degraded, the archaea proteins are highly valued in biotechnology for their stability and ability to function. Currently, the archaea domain is categorized into three main subgroups, at the phylum level: euryarchaeota, crenarchaeota, and thaumarchaeota [3]. Archaeal organisms have diverse distinguishing characteristics, including unique cell wall and membrane components, distinct metabolic pathways, and enzymes, whereas other features are shared either with bacteria or with eukaryotes [4]. Hyperthermophilic archaea such as *Aeropyrum pernix* and *Pyrococcus horikoshii* growing optimally at≥90°C produce many thermophilic enzymes including phospholipase.

Phospholipases are enzymes that cleave the various bonds in phospholipids. These enzymes are various in the active site, physiological function, mode of work, and their regulation [5]. According to the position of bond cleavage in their substrates, they are classified into five groups: A1, A2, B, C, and D [6]. Phospholipases, as versatile biocatalysts are commonly utilized in several industries, for instance, oil degumming, food, detergents, nutraceuticals, biodiesels, agriculture, bioremediation, leather, paper, and cosmetics [7-9].

The field of machine learning employs the utilization of past data to facilitate the development of a predictive model tailored for the projection of future data. The proliferation of data in contemporary biology has led to the growing prevalence and versatility of machine learning algorithms for categorizing, predicting, and grouping biological data through the methods of clustering, regression, and classification [10, 11]. Support vector machine (SVM) stands out as a potent machine learning algorithm, capable of predicting the class labels of unknown data by leveraging an effective model that is derived from training data [12]. The condition in exerting the SVM algorithm is that each class member to be identified must be available for training. The principle of SVM is that attempts to find the most suitable separating hyperplane for given datasets which are assumed as points in a high-dimensional space. Afterward, based on the placement of an unknown dataset on each side of the learned hyperplane, SVM can predict the status of the dataset [13]. An amended version of KNN algorithm, Optimized Evidence-Theoretic K-Nearest Neighbor (OET-KNN) is an algorithm based on the Dempster–Shafer theory [14]. K-NN is a nonparametric classification model and works based on a majority voting mechanism in that for detecting the class of input data, the nearest neighbor class is selected among the found k neighbors [14]. Random Forest (RF) consists of randomly generated decision trees constructed from a training dataset. Each tree predicts a discrete class, then the class of the test data is identified by the class with a greater predicted number among the trees [15]. Covariance Discriminant (CD) is derived from Mahalanobis distance discriminant with the difference that it applies some corrections to reduce the effect of the imbalanced data set on the prediction results [17].

There are various methodologies aimed at predicting diverse protein characteristics; however, a large number of these approaches emphasize the analysis of amino acid composition [16, 17], sequence [18, 19], and template. In this research, we have utilized the concept of Chou’s pseudo amino acid composition (PseAAC) to predict archaeal phospholipase enzymes. PseAAC displays a protein sequence with a distinct model without thoroughly losing the information behind its sequence [20]. Originally, it was developed by Chou in 2001 for predicting the protein subcellular localization and membrane protein types [21, 22]. 

## MATERIALS AND METHODS

**Dataset Selection: **The positive dataset for this study was obtained from the National Center for Biotechnology Information (NCBI) database, which contained 835 archaeal protein sequences of phospholipase. In addition, a negative dataset was also collected from the same source, consisting of 711 sequences of non-phospholipase proteins. To safeguard the quality of the datasets, sequences that were deemed putative, partial, or fragmental were excluded from consideration. The ExPASy website's Decrease Redundancy tool was utilized to ensure optimal data quality in our datasets. Specifically, sequences exhibiting below 90% similarity were retained to prevent any potential classifier bias. The final positive dataset was including 493 sequences. Also, negative dataset sequences for archaeal non-phospholipase proteins were decreased to 526 sequences.

**BioSeq-Analysis server: **In this research, we used the BioSeq-Analysis server available at http://bioinformatics.hitsz.edu.cn/BioSeq-Analysis/ which can do automatically the following three main steps: selecting features, constructing a predictor, and evaluating the performance of the predictor. BioSeq-Analysis is constructed from three sub-servers, DNA-Analysis, RNA-Analysis, and Protein-Analysis. This web server is a vigorous platform for the analysis of biological sequence orders based on machine learning algorithms [23]. 

**Protein-Analysis sub-server:** Protein-Analysis sub-server was chosen and the following three main steps were performed:

**Extracting features: **Various PseAAC modes including PC-PseAAC, SC-PseAAC, PC-PseAAC-General, and SC-PseAAC-General were applied to extract features. For generating different kinds of PseAAC, the values of the amino acid physicochemical properties, weight factor (w), and correlation rank (λ) were utilized.

PseAAC of a sample protein is represented by a set of $20^{+\lambda }$ discrete factors. The first 20 ones illustrate the conventional AAC components and the λ factor represents the sequence order correlation and incorporated physiochemical features [20, 24].

The present study employs PC-PseAAC and SC-PseAAC models, which integrate amino acid composition and global sequence-order influences using parallel and series correlation mechanisms to generate characteristic protein vectors. The PC-PseAAC-General and SC-PseAAC-General algorithms, which incorporate 547 physicochemical properties extracted from the amino acid index, are augmented with more complex information, such as functional dom-ain (FunD), sequential evolution, gene ontology (GO), and other customizable properties [25]. In this study, for PC-PseAAC and SC-PseAAC modes, hydrophobicity, hydrophilicity and mass were selected, while for PC-PseAAC-General and SC-PseAAC-General, in addition to the three above-mentioned properties, five additional physicochemical properties from the first line were selected. In order to prioritize the incorporation of supplementary pseudo components over traditional sequence components, the weight factor (ω) has been developed [26]. Moreover, the Lambda parameter (λ) represents the correlation counted rank along a protein sequence. Lambda must be adjusted to a positive integer (such as 0, 1, 2) and smaller than L-k, where L is the query sequence length and k is the length of the selected oligomer mode [27]. In this study, λ and ω parameters were optimized.

**Constructing a predictor:** Machine learning algorithms such as OET-KNN, RF, SVM, and CD were applied for constructing predictors.

**Evaluating the performance of the predictor: **The efficacy of the developed predictors was assessed through the utilization of the 5-fold cross-validation and bootstrapping methods. The 5-fold cross-validation method involves partitioning the input data set into five distinct sub-datasets, of equal size, in a random manner. Two sub-datasets are designated as the validation and test sets, while the remaining three sub-datasets are categorized as training sets. Optimization of parameters is executed utilizing the validation set, while evaluation of the overall system performance is accomplished through the utilization of the test set. The procedure is iterated a total of five times to ensure that every sub-dataset serves as the test set at least once [30]. In the context of bootstrapping, the benchmark dataset was subjected to 20 random samplings, and the ultimate outcomes were subsequently derived from the collective mean value of these samplings [31]. The effectiveness evaluation was measured via five parameters: accuracy (Acc), specificity (Sp), and sensitivity (Sn), Matthews’s correlation coefficient (MCC), and area under the receiver operating characteristics (ROC) curve (AUC). Furthermore, the ROC curve was generated. 

Acc, Sp, and Sn were calculated according to (Eqs. 1-3) and MCC was calculated according to (Eq. 4) that is considered as a balanced measure in which the TP, TN, FP, and FN are taken into account. 

 Acc = (TP+TN)/(TP+TN+FP+FN) (1) 

 Sp = TN/(TN+FP) (2) 

 Sn = TP/(TP+FN) (3)

 MCC = ((TP*TN) – (FP*FN))/√((TP+FP)(TP+FN)(TN+FP)(TN+FN)) (4)

Where, the abbreviation TP represents the metric of True Positive, which corresponds to the numerical value indicating the total count of positive sequences that are correctly identified as positive by a particular model or algorithm. The term "FP" refers to False Positive, which signifies the number of negative sequences that are erroneously identified as positive. TN refers to True Negatives, denoting the number of negative sequences that have been accurately classified as negative. FN denotes False Negative, which signifies the number of positive occurrences that are characterized as negative [26, 28]. 

The variable 'Acc' quantifies the count of sequences which have been accurately classified out of the entire set of sequences. This term denotes the accuracy of the classification system utilized in the context. The metric referred to as "Sp" in the context of algorithmic performance evaluation measures the accuracy with which negative data is predicted by the system, specifically with regard to all actual instances of negative sequences. This statistical measure is commonly known as the true negative rate. Thus, outcomes that exhibit a substantial degree of specificity are dependable in academic discourse. Similarly, the sensitivity (Sn) of a classifier is indicative of the true positive sequences predicted as positive. A high Sn value depicts positive predicted outcomes that are dependable and accurate. The utilization of MCC is commonly applied in the assessment of binary classification. The acceptable range for MCC values is continuous and falls within the interval of -1 to +1. A perfect prediction is indicated by a value of +1, a value of 0 suggests a random prediction and a value of -1 represents the absolute discrepancy between the predicted outcome and the observed result. A classifier with high Sp, Sn, and ACC values (approximately more than 70–80%) and an MCC of near +1 is reliable [33, 34]. 

A ROC curve visualizes the effectiveness of classifiers by a two-dimensional depiction. In this curve, the y-axis represents the TP rate and the x-axis shows the FP rate. AUC is defined as the area under the curve in the unit square, and its value is always between 0 and 1.0 [29]. 

## RESULTS

In this study, three steps were performed via the BioSeq-Analysis server. Different PseAAC modes were applied. Calculations by BioSeq-Analysis for some of the physicochemical properties were considered. Two parameters, λ and w, were optimized. For the analysis of data, OET-KNN, RF, SVM and CD classifiers were applied to the dataset. The 5-fold cross-validation and bootstrapping were carried out to figure out the performance of the predictors.

The results of PC-PseAAC, PC-PseAAC-General, SC-PseAAC, and SC-PseAAC-General modes are summarized in Tables 1, 2, 3 and 4. All four machine learning algorithms had an excellent total accuracy of >= 90% for 5-fold cross-validation, and >= 88% for bootstrapping, certifying the provided results. 

According to 5-fold cross-validation: OET-KNN presented the highest values of Acc (95%), Sn (95%) and MCC (0.90). However, the highest specificity (97%) was obtained by RF algorithm. Additionally, in both OET-KNN and RF algorithms, the highest value of AUC (0.98) was achieved. Among the algorithms, the lowest values for Acc (90%), Sp (91%), Sn (89%), MCC (0.80) and the lowest value for AUC (0.09) was attained by SVM and CD algorithms, respectively. 

According to bootstrapping: OET-KNN and RF presented the highest values of Acc (93%), MCC (0.87) and AUC (0.98). The highest Sn (94%) and Sp (95%) were provided by OET-KNN and RF, respectively. The lowest value of AUC was provided by CD classifier. Table 1 shows the details of the results provided by each algorithm.

**Table 1:** The performance of the classifiers by PC-PseAAC mode with 5-fold cross validation and bootstrapping

**Validation Test** **Algorithm**	**5- fold cross validation**				**Bootstrapping**			
	**SVM**	**RF**	**OET-KNN**	**CD**	**SVM**	**RF**	**OET-KNN**	**CD**
**Accuracy %**	90	94	**95**	92	90	**93**	**93**	90
**Specificity %**	91	**97**	95	93	91	**95**	93	94
**Sensitivity %**	89	92	**95**	92	89	91	**94**	85
**MCC**	0.8	0.89	**0.9**	0.85	0.8	**0.87**	**0.87**	0.8
**AUC**	0.96	**0.98**	**0.98**	0.09	0.95	**0.98**	**0.98**	0.11
**λ**	7	7	8	8	8	7	8	8
**w**	0.1	0.1	0.1	0.7	0.1	0.1	0.1	0.7

**Figure 1:**
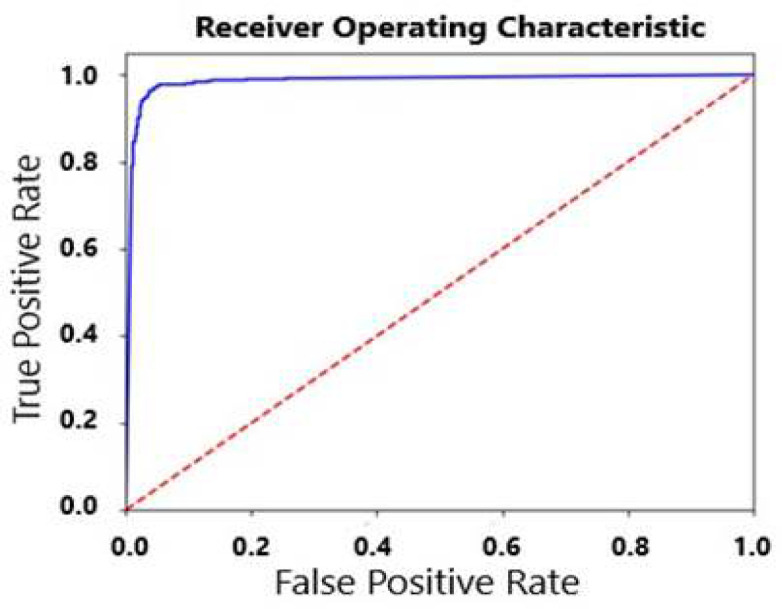
ROC curve of OET-KNN classifier and SC-PseAAC mode with AUC of 0.98.

According to 5-fold cross-validation: The OET-KNN provided the highest values of Acc (96%), Sn (96%), and MCC (0.92). Although OET-KNN, RF and SVM showed a similarly high value for AUC (0.98), CD showed the lowest one (0.11) (Fig. 1). The lowest value for Acc (92%), Sn (87%), and MCC (0.86), but the highest Sp (97%) belonged to CD.

According to bootstrapping: Similar to the 5-fold cross-validation result, the OET-KNN presented the highest values in Acc (95%), Sn (96%), and MCC (0.90). Also,the highest Sp was provided by the CD (98%). CD presented the lowest value of AUC while the others had a similar value of AUC (0.98). Among the algorithms, the lowest value for Acc (88%), Sn (78%), and MCC (0.78) belonged to CD. The detailed results for the performed predictions are provided in Table 2.

**Table 2:** The performance of the classifiers by SC-PseAAC mode with 5-fold cross validation and bootstrapping

**Validation Test** **Algorithm**	**5- fold cross validation**				**Bootstrapping**			
	**SVM**	**RF**	**OET-KNN**	**CD**	**SVM**	**RF**	**OET-KNN**	**CD**
**Accuracy %**	95	94	**96**	92	94	94	**95**	88
**Specificity %**	95	96	95	**97**	94	96	94	**98**
**Sensitivity %**	95	92	**96**	87	94	92	**96**	78
**MCC**	0.9	0.88	**0.92**	0.86	0.89	0.88	**0.9**	0.78
**AUC**	**0.98**	**0.98**	**0.98**	0.11	**0.98**	**0.98**	**0.98**	0.13
**λ**	9	6	8	4	9	8	8	4
**w**	0.3	0.1	0.1	0.1	0.3	0.3	0.1	0.1

According to 5-fold cross-validation: Similar to PC-PseAAC result with 5-fold cross-validation, OET-KNN algorithm showed the highest values for the classification including; Acc (95%), Sn (94%) and MCC (0.90). Moreover, the highest value of AUC (0.98) was provided by both OET-KNN and RF algorithms. The lowest Acc, Sp, and MCC value was presented by SVM algorithm and the lowest Sn and AUC value was provided by CD.

According to bootstrapping: OET-KNN and RF presented the highest values of Acc (93%) and MCC (0.87). The highest Sn (94%), Sp (97%) and AUC (0.98) were provided by OET-KNN, CD and RF, respectively. CD classifier gave the lowest values of Acc, Sn, MCC and AUC. The lowest value of Sp and MCC were obtained by SVM. The detailed results for the performed prediction are provided in Table 3.

**Table 3:** The performance of the classifiers by PC-PseAAC-General mode with 5-fold cross validation and bootstrapping

**Validation Test** **Algorithm**	**5- fold cross validation**				**Bootstrapping**			
	**SVM**	**RF**	**OET-KNN**	**CD**	**SVM**	**RF**	**OET-KNN**	**CD**
**Accuracy %**	90	94	**95**	91	89	**93**	**93**	88
**Specificity %**	91	**96**	95	**96**	91	95	92	**97**
**Sensitivity %**	89	92	**94**	86	88	92	**94**	79
**MCC**	0.8	0.88	**0.9**	0.84	0.79	**0.87**	**0.87**	0.79
**AUC**	0.95	**0.98**	**0.98**	0.1	0.95	**0.98**	0.97	0.12
**λ**	9	3	3	8	9	3	3	8
**w**	0.3	0.3	0.1	0.1	0.3	0.5	0.1	0.1

According to 5-fold cross-validation: The highest values in Acc (96%) and MCC (0.92) were provided by the SVM model, but the highest Sp (98%) and Sn (96%) were provided by the CD and OET-KNN, respectively. For the AUC value, CD had the lowest value and the three others had a similarly high value of 0.98. Among the applied algorithms, the lowest value for Acc (92%), Sn (85%), and MCC (0.85) belonged to the CD classifier. 

According to bootstrapping: Like previous 5-fold cross-validation results, the SVM model presented the highest values in Acc (95%) and MCC (0.90), but the highest Sp (98%) and Sn (96%) were provided by the CD and OET-KNN, respectively. CD model presented the lowest value of AUC while the three others gave a similar value of AUC (0.98). Among the algorithms, the lowest value for Acc (90%), Sn (80%), and MCC (0.81) belonged to CD. The detailed results for the performed prediction are provided in Table 4.

**Table 4:** The performance of the classifiers by SC-PseAAC-General mode with 5-fold cross validation and bootstrapping

**Validation Test** **Algorithm**	**5- fold cross validation**				**Bootstrapping**			
	**SVM**	**RF**	**OET-KNN**	**CD**	**SVM**	**RF**	**OET-KNN**	**CD**
**Accuracy %**	**96**	94	95	92	**95**	94	94	90
**Specificity %**	97	96	95	**98**	96	95	93	**98**
**Sensitivity %**	95	91	**96**	85	94	92	**95**	80
**MCC**	**0.92**	0.88	0.91	0.85	**0.9**	0.88	0.88	0.81
**AUC**	**0.98**	**0.98**	**0.98**	0.09	**0.98**	**0.98**	**0.98**	0.14
**λ**	8	6	6	3	8	3	6	3
**w**	0.9	0.1	0.1	0.1	0.9	0.1	0.1	0.1

## DISCUSSION

Machine learning computer programs are used to find meaningful patterns in data. The practical implementation of machine learning has garnered widespread attention in scientific disciplines including bioinformatics and medicine [36]. One of its applications is data classification such as classification of the large datasets of various enzyme molecules [37].

Phospholipases refer to a class of lipolytic enzymes that specifically catalyze the hydrolysis of ester bonds in phospholipid substrates, and are characterized by their broad range of functional applications [30]. Archaea are a cohort of life forms that bear resemblance to bacteria, yet distinguish themselves through their unique evolutionary lineage. Numerous instances of archaeal organisms inhabiting extreme environments, such as areas characterized by elevated pressures, salt concentrations, or temperatures, have been uncovered through scientific inquiry. In recent years, there has been a growing interest in the potential industrial applications of thermostable phospholipases derived from archaea [31]. Due to the significant value of archaeal phospholipases, it is imperative to undertake the task of anticipating and categorizing them from other enzymes. Therefore, it would be of great benefit to gather data regarding the effectiveness of various machine learning algorithms, which could facilitate further exploration of this enzyme and aid in the creation of a server for data classification [14]. In the present investigation, a set of machine learning algorithms, for example, OET-KNN, RF, SVM, and CD were employed along with two evaluation tests to investigate the data analysis and interpretation of PseAAC.

Chou’s PseAAC [38] has emerged as a powerful technique for protein categorization. To avoid the complete deprivation of sequence-pattern data for proteins, PseAAC [26] has been developed.

Different types of PseAAC are employed to predict protein structural class [32], bacterial secreted proteins [33], cyclins [34], risk type of human papillomaviruses [35], enzyme subfamily classes [24, 36, 37], G-protein coupled receptor classes [38-40], cell wall lytic enzymes [41], subcellular localization of apoptosis proteins [42, 43], lipase types [44], subcellular localization of mycobacterial proteins [45], cofactors of oxidoreductases [46], DNA-binding proteins [47], quaternary structural attributes [48], proteases and their types [49] GABAA receptors [50] and Glutathione S-transferases [51-53].

BioSeq-Analysis, a platform established in 2017, is introduced for the primary purpose of analyzing diverse DNA, RNA, and protein sequences at the sequence level, utilizing machine learning techniques and diverse modes, including distinct varieties of PseAAC and Kmer. BioSeq-Analysis is increasingly applied in many areas of computational biology [25].

According to the results provided in this research, we can notice that based on three types of PseAAC, OET-KNN algorithm had the highest accuracy in both performance evaluation tests, however SVM had the highest accuracy in just one type of PseAAC. It is interpreted from very high values of accuracy (96%), specificity (95%), sensitivity (96%), MCC (0.92) and AUC (0.98) obtained by OET-KNN (in SC-PseAAC mode and 5-fold cross-validation) that OET-KNN predictor is a powerful machine learning algorithm for the classification of enzymes as phospholipase or non-phospholipase. MCC value of 0.92 confirms the significant ability of OET-KNN in prediction and AUC value of 0.98, near to 1, means that OET-KNN is a realistic classifier and its result is very reliable.

In Shen and Chou's investigation, OET-KNN classifier and PseAAC method were utilized to predict membrane protein types. The Overall rates of correct prediction obtained by OET-KNN and PseAAC were 99.5, 84.7 and 94.2 % in self-consistency, jackknife, and independent dataset tests, respectively. These values were higher than those obtained by other approaches. OET-KNN classifier may have a positive impact in improving the prediction quality for many other protein attributes, such as protein structural class, protein subcellular localization, enzyme family and subfamily class, G-protein coupled receptor type, and protein quaternary structure types. 

 In our study, OET-KNN achieved a very high accuracy, because the OET-KNN rule obtained through an optimization treatment could lead to a substantial improvement in classification accuracy and improve prediction quality. 

Currently, there exists no anticipatory server for archaeal phospholipases in the academic literature. The present study illustrates that the utilization of Chou's PseAAC and OET-KNN models is an efficient approach for the anticipation of phospholipases in archaea.

## Conflict of Interest:

There are no conflicts of interest associated with this manuscript.
